# Thyroid Function Tests and Serum Amylase Levels in Chronic Kidney Disease: A Cross-Sectional Study Correlating Biochemical Changes With Disease Severity at a Tertiary Care Centre in India

**DOI:** 10.7759/cureus.104974

**Published:** 2026-03-10

**Authors:** Pranav Sharma, Anil Samaria, Harsh Tak, Monika Chowdhary, Mayank Shrivastava, Shubham Maharia

**Affiliations:** 1 Internal Medicine, Jawaharlal Nehru Medical College, Ajmer, IND

**Keywords:** chronic kidney disease, estimated glomerular filtration rate, serum amylase, subclinical hypothyroidism, thyroid function tests

## Abstract

Background: Chronic kidney disease (CKD) represents a major and growing health concern in both global and Indian populations. In addition to progressive renal dysfunction, CKD is frequently accompanied by metabolic and endocrine disturbances that may affect disease progression and patient outcomes. Alterations in thyroid hormone and serum amylase levels are frequently encountered in CKD patients, even in the absence of overt thyroid or pancreatic disease, and may hold important diagnostic and prognostic relevance.

Aim: The aim of this study was to evaluate thyroid function parameters (free triiodothyronine (FT3), free thyroxine (FT4), thyroid-stimulating hormone (TSH)) and serum amylase levels in patients with CKD, and to assess their association with disease severity.

Methods: This hospital-based cross-sectional observational study was conducted at a tertiary care centre in India. Adult patients with CKD were classified into stages G1-G5 based on estimated glomerular filtration rate (eGFR). Thyroid function tests were measured using chemiluminescent immunoassay, while serum amylase was estimated by enzymatic colorimetric methods. Statistical analyses were performed to compare biochemical parameters across CKD stages and to evaluate correlations with renal function.

Results: Significant differences were observed in FT3 and FT4 levels across CKD stages (p=0.010 and p=0.003, respectively), with lower values in advanced stages. TSH levels showed a significant increasing trend across stages (p=0.029). Serum amylase levels demonstrated a rising trend in advanced CKD; however, the difference did not reach statistical significance (p=0.12).

Conclusion: Thyroid dysfunction and alterations in serum amylase levels are common findings in patients with CKD and show meaningful associations with disease severity. Incorporating endocrine and enzymatic evaluation alongside conventional renal markers may provide additional clinical insight in the evaluation of patients with CKD.

## Introduction

Chronic kidney disease (CKD): definition and staging

CKD is characterised by a persistent and progressive decline in renal function that progresses over time and is present for more than three months. CKD poses a significant burden on healthcare systems worldwide. The disease is associated with increased morbidity, mortality, and substantial socioeconomic impact, particularly in low- and middle-income countries. Based on estimated glomerular filtration rate (eGFR), CKD is classified into five stages, ranging from mild functional impairment to end-stage renal disease (ESRD), requiring renal replacement therapy [1]. The Kidney Disease Outcomes Quality Initiative (KDOQI) and subsequent Kidney Disease: Improving Global Outcomes (KDIGO) guidelines provide standardized criteria for evaluation, classification, and stratification of CKD severity [2].

Epidemiology of CKD

CKD is a significant global cause of morbidity and mortality, with reported prevalence estimates of 10-15% [3]. According to data from the Global Burden of Disease (GBD) collaboration, CKD ranks among the leading causes of death worldwide, and its contribution is expected to rise further over the coming decades, as highlighted in the GBD CKD Collaboration report (2020) [4]. In the Indian context, the SEEK (Screening and Early Evaluation of Kidney Disease) study documented a prevalence of approximately 17%, with a high proportion of patients diagnosed at advanced stages due to limited awareness and insufficient screening practices [5]. While clinical studies establish CKD as a highly prevalent chronic condition, emerging forensic evidence has highlighted its substantial mortality impact. A recent study reported the presence of CKD in 25.4% of sudden adult deaths, underscoring its potential contribution to fatal outcomes [6]. Collectively, these observations underscore CKD as a growing yet under-recognized public health concern, particularly in low- and middle-income countries.

Systemic complications of CKD

CKD is not merely a renal disorder but a systemic condition associated with multiple complications. Cardiovascular disease remains the leading cause of mortality, influenced by hypertension, oxidative stress, and vascular calcification [7]. Endocrine abnormalities such as anemia due to erythropoietin deficiency and disturbances in mineral metabolism leading to renal osteodystrophy are also frequent [8]. Metabolic derangements, including dyslipidemia and acidosis, contribute further to disease progression and increased mortality [9].

Thyroid dysfunction in CKD

Thyroid dysfunction is a well-documented but often under-recognized complication of CKD. The most common abnormality is the low T3 syndrome, characterized by decreased serum triiodothyronine (T3), with normal or reduced thyroxine (T4) and variable thyroid-stimulating hormone (TSH) levels [10,11]. This is attributed to reduced peripheral conversion of T4 to T3, impaired hormone binding, and altered iodine metabolism [12]. In advanced stages, subclinical or overt hypothyroidism may be observed, further complicating metabolic and cardiovascular outcomes [13].

Serum amylase alterations in CKD

Serum amylase levels are frequently altered in CKD due to impaired renal clearance of pancreatic and salivary isoenzymes [14]. This elevation often occurs in the absence of pancreatitis, complicating interpretation in uremic patients [15,16]. Previous studies suggest that while amylase levels tend to rise with declining renal function, their diagnostic specificity is limited in CKD patients [17].

Rationale and study justification

Although several international studies have explored thyroid dysfunction and serum amylase alterations in CKD, data from the Indian population remain limited and heterogeneous. Variations in demographic profile, nutritional status, healthcare access, and late-stage presentation may influence biochemical patterns in Indian CKD patients. A tertiary care center-based evaluation allows assessment across advanced disease stages and provides clinically relevant insights applicable to routine nephrology practice in resource-constrained settings.

Aims and objectives

The primary aim of this study was to evaluate thyroid function parameters, including free T3 (FT3), free T4 (FT4), TSH, and serum amylase levels among patients with CKD. The study also aims to examine the association between these biochemical parameters and the severity of CKD.

## Materials and methods

Study design and setting

This was a hospital-based prospective cross-sectional study conducted in the Department of Medicine at Jawaharlal Nehru Medical College and Hospital, Ajmer, a tertiary care centre catering to patients from Rajasthan and neighbouring regions in India. The study was conducted from July 2023 to December 2024.

Study population

Adult patients diagnosed with CKD presenting to the department (both outpatient and inpatient) during the study period were consecutively included and prospectively evaluated for thyroid function tests and serum amylase levels. Patients meeting the inclusion criteria were approached for participation, and written informed consent was obtained prior to enrollment. Biochemical investigations, including thyroid function tests and serum amylase levels, were performed as part of routine clinical evaluation. No additional financial burden was imposed on participants for study-related testing. A total of 225 patients diagnosed with CKD were enrolled and classified into stages G1-G5 based on eGFR, calculated using the Cockcroft-Gault equation [18], consistent with the institutional laboratory protocol and routine clinical practice at our centre during the study period. It is acknowledged that the CKD-EPI equation is now more widely recommended for staging.

Inclusion Criteria

Inclusion criteria were adult patients aged 18-65 years with a diagnosis of CKD, categorized into stages G1-G5 according to eGFR.

Exclusion Criteria

Given the known limitations of the Cockcroft-Gault equation and age-related variations in renal and thyroid function in elderly individuals, patients aged above 65 years were excluded to enhance staging consistency and minimize confounding. Patients with pre-existing thyroid disorders, hypothalamic-pituitary axis abnormalities, pregnancy, acute pancreatitis, mumps, recent endoscopic retrograde cholangiopancreatography (ERCP), burn injuries, pancreatic or salivary gland disorders, and those on medications known to significantly alter thyroid function (e.g. amiodarone, lithium, glucocorticoids, interferon therapy, antithyroid drugs) or to influence serum amylase levels (e.g. valproate, thiazide diuretics, azathioprine, corticosteroids) were excluded.

Medication history was obtained through a detailed patient interview and review of medical records. The presence of exclusion comorbidities was determined based on detailed clinical history, review of prior medical records, medication history, and relevant laboratory and imaging investigations where available. Patients with previously documented diagnoses or ongoing treatment for thyroid disorders, pancreatitis, or other specified conditions were excluded accordingly.

Sample size calculation

The sample size was calculated using Fisher's z-transformation to detect a moderate correlation (r=0.25) between eGFR and the studied biochemical parameters, with 95% confidence level and 80% statistical power. The minimum required sample size was approximately 124 participants. However, given the planned subgroup analyses across CKD stages (G1-G5) and to enhance the precision and stability of correlation estimates, a larger sample was targeted. Accordingly, 225 patients were consecutively enrolled during the study period.

Assessment of renal function and CKD staging

Renal function was evaluated by estimating eGFR using the Cockcroft-Gault equation [18]. Based on the calculated eGFR values, patients were categorized into CKD stages as follows: (i) Stage 1: Kidney damage with normal or increased GFR (>90 mL/minute/1.73 m^2^), (ii) Stage 2: Mild reduction in GFR (60-89 mL/minute/1.73 m^2^), (iii) Stage 3a: Moderate reduction in GFR (45-59 mL/minute/1.73 m^2^), (iv) Stage 3b: Moderate reduction in GFR (30-44 mL/minute/1.73 m^2^), (v) Stage 4: Severe reduction in GFR (15-29 mL/minute/1.73 m^2^), (vi) Stage 5: End stage (GFR<15 mL/minute/1.73m^2^).

Data collection

Relevant clinical information was obtained from patient interviews and hospital medical records. Laboratory evaluation included blood urea and serum creatinine to calculate eGFR, thyroid function tests (FT3, FT4, and TSH), and serum amylase levels. These parameters were recorded to assess biochemical alterations across different stages of CKD.

Laboratory Methods

Venous blood samples were obtained under aseptic conditions for the assessment of thyroid function parameters and serum amylase levels. Thyroid function evaluation included serum FT3, FT4, and TSH, which were measured using a chemiluminescent immunoassay on a fully automated analyser. Serum amylase levels were estimated using an enzymatic colorimetric method.

Statistical analysis

Data analysis was performed using IBM SPSS Statistics for Windows, version 25.0 (Released 2017; IBM Corp., Armonk, New York, United States). Continuous variables were presented as mean ± standard deviation (SD), whereas categorical variables were expressed as frequencies and percentages. Differences in biochemical parameters across CKD stages using one-way analysis of variance (ANOVA), with statistical significance assessed using corresponding p-values. A p-value of less than 0.05 was considered statistically significant.

Ethical considerations

The study was approved by the Institutional Ethics Committee of Jawaharlal Nehru Medical College, Ajmer (approval number: 2429/Acad-III/MCA/2023, dated August 31, 2023). Written informed consent was obtained from all participants before enrolment.

## Results

Demographics

A total of 225 patients were included in the study, of which 54.2% were male and 45.8% were female, as shown in Table 1. Table 2 presents the distribution of the study population across various stages of CKD as classified by eGFR. A total of 225 patients were included, with the largest proportions observed in stage G4 and stage G5 (22.2% each), while stage G1 accounted for the smallest proportion of patients (8.9%). As the primary objective of the present study was to evaluate biochemical parameters (thyroid function tests and serum amylase levels) in relation to CKD stages, additional demographic or comorbidity variables were not included in the detailed analyses.

**Table 1:** Distribution of study population according to sex (N=225)

Sex	Number of Patients (Percentage)
Male	122 (54.2%)
Female	103 (45.8%)

**Table 2:** Distribution of study population by CKD stages (N=225) CKD: chronic kidney disease; eGFR: estimated glomerular filtration rate

CKD Stage (eGFR Range)	Number of Patients (Percentage)	eGFR, mean ± SD
G1 (≥ 90 mL/min/1.73m^2^)	20 (8.9%)	100 ± 6.52 mL/min/1.73m^2^
G2 (60-89 mL/min/1.73m^2^)	30 (13.3%)	73.79 ± 8.10 mL/min/1.73m^2^
G3a (45-59 mL/min/1.73m^2^)	40 (17.8%)	51.62 ± 4.48 mL/min/1.73m^2^
G3b (30-44 mL/min/1.73m^2^)	35 (15.6%)	37.88 ± 4.06 mL/min/1.73m^2^
G4 (15-29 mL/min/1.73m^2^)	50 (22.2%)	21.4 ± 3.68 mL/min/1.73m^2^
G5 (<15 mL/min/1.73m^2^)	50 (22.2%)	8.57 ± 3.06 mL/min/1.73m^2^

Thyroid profile

Thyroid function analysis revealed stage-dependent changes, as shown in Table 3, which demonstrates the thyroid function parameters across different CKD stages. Although FT3 levels appeared relatively higher in intermediate stages, an overall decline was observed in advanced CKD (G4-G5) (p = 0.010). The relatively lower value in G1 may reflect a smaller sample size in this subgroup and baseline variability. FT4 levels demonstrated a modest declining trend in later stages (p = 0.003), while TSH levels progressively increased with worsening renal function (p=0.029). This hormonal pattern is consistent with changes observed in subclinical hypothyroidism or non-thyroidal illness syndrome in CKD [10,11].

**Table 3:** Thyroid profile trends across CKD stages One-way analysis of variance (ANOVA), performed using stage-wise means, standard deviations, and sample sizes, demonstrated significant differences in FT3 levels across CKD stages; F(5,219)=3.08, p=0.010. A similar significant variation was observed for FT4 levels; F(5,219)=3.63, p=0.003. TSH levels also differed significantly across stages, though with a smaller effect size; F(5,219)=2.54, p=0.029. CKD: chronic kidney disease; FT4: free thyroxine; FT3: free triiodothyronine; TSH: thyroid-stimulating hormone

Parameter	G1 (n=20)	G2 (n=30)	G3a (n=40)	G3b (n=35)	G4 (n=50)	G5 (n=50)	P Value
FT3 (pg/mL), mean ± SD	1.2 ± 0.74	2.07 ± 1.42	2.45 ± 0.83	2.21 ± 0.63	2.11 ± 0.72	1.98 ± 0.69	0.010
FT4 (ng/dL), mean ± SD	0.16 ± 0.29	0.79 ± 0.14	0.79 ± 0.23	0.79 ± 0.31	0.68 ± 0.28	0.67 ± 0.28	0.003
TSH (μIU/mL), mean ± SD	2.56 ± 3.20	3.74 ± 2.84	3.96 ± 2.75	3.20 ± 2.45	5.41 ± 2.97	4.31 ± 3.17	0.029

Serum amylase

While serum amylase levels demonstrated an overall increasing trend from 108 IU/L in stage G1 to 148.6 IU/L in stage G5, as shown in Table 4, this difference did not reach statistical significance (p=0.129).

**Table 4:** Serum amylase levels across CKD stages One-way analysis of variance (ANOVA), performed using stage-wise means, standard deviations, and sample sizes, did not demonstrate a statistically significant difference in serum amylase levels across CKD stages; F(5,219)=1.73, p=0.129. Although higher mean values were observed in advanced stages, this trend did not reach statistical significance. CKD: chronic kidney disease

Parameter	G1 (n=20)	G2 (n=30)	G3a (n=40)	G3b (n=35)	G4 (n=50)	G5 (n=50)	P Value
Serum Amylase (U/L), mean ± SD	108 ± 41.88	118.67 ± 17.01	127 ± 41.88	117.13 ± 33.69	125.83 ± 43.40	148.58 ± 49.51	0.129

## Discussion

The present study evaluated demographic characteristics, thyroid function parameters, and serum amylase levels across different stages of CKD and examined their association with disease severity.

The study population demonstrated a male predominance, as shown in Table 2. Distribution across CKD stages revealed a higher representation of patients in advanced stages (G4 and G5), as detailed in Table 1. A relatively higher proportion of female patients was observed in stage G5 compared to earlier CKD stages (data not shown). Similar demographic shifts with disease severity have been reported in earlier Indian CKD cohorts [5].

Thyroid function abnormalities constituted one of the principal findings of this study. A significant variation in FT3 and FT4 levels, with an overall decline in advanced CKD stages (Table 3), along with a concomitant rise in TSH, was observed with declining renal function (eGFR). This pattern reflects the characteristic low T3 syndrome and a shift toward subclinical hypothyroidism. These alterations have been attributed to impaired peripheral conversion of T4 to T3, altered thyroid hormone binding, and disturbances in iodine metabolism in uremic states [10,11]. Importantly, thyroid dysfunction in CKD is clinically relevant, as subclinical hypothyroidism has been associated with increased cardiovascular risk and adverse outcomes in this population. These changes may influence overall prognosis rather than representing purely adaptive responses [19].

Serum amylase levels were higher in advanced CKD stages compared with earlier stages, although the difference was not statistically significant (Table 4). This observation is consistent with previous studies attributing hyperamylasemia in CKD primarily to reduced renal clearance of pancreatic and salivary isoenzymes rather than true pancreatic pathology [14,15]. Awareness of this phenomenon is clinically important, as elevated serum amylase levels in CKD patients may lead to diagnostic confusion with acute pancreatitis if interpreted without appropriate clinical correlation [16,17]

The present study demonstrates stage-related alterations in thyroid function parameters across CKD stages, along with a non-significant trend toward higher serum amylase levels in advanced disease. These findings suggest that endocrine and enzymatic changes may accompany worsening renal function [20]. However, given the cross-sectional design, these associations should be interpreted cautiously. Larger longitudinal studies are required to clarify their clinical implications.

Although serum amylase levels showed a rising trend in advanced CKD stages, the difference did not reach statistical significance. Therefore, no definitive association between serum amylase level and CKD severity can be concluded from the present study. Further studies with larger sample sizes are warranted to clarify this relationship. 

Limitations and future recommendations

This study has several limitations. First, its single-center cross-sectional design limits generalizability and precludes causal inference. Second, the sample size within individual CKD subgroups may have limited statistical power for certain comparisons. Third, eGFR was calculated using the Cockcroft-Gault formula, which may be influenced by variations in age, body weight, and muscle mass, potentially affecting accuracy across different patient populations. Fourth, potential confounding variables such as comorbid conditions (e.g., diabetes mellitus, hypertension), medication use, and nutritional status were not adjusted for in the analysis. Additionally, longitudinal follow-up data were not available to assess prognostic implications. Future multicenter prospective studies with larger sample sizes and multivariable adjustment are required to validate these findings and determine their clinical relevance.

## Conclusions

The present study demonstrates that patients with CKD exhibit progressive alterations in thyroid function, characterized by alterations in FT3 and FT4 levels with an overall decline in advanced CKD stages and a corresponding rise in TSH. Although serum amylase levels showed a gradual increase across advancing CKD stages, this difference did not reach statistical significance. Therefore, while impaired renal clearance may theoretically contribute to altered amylase levels in CKD, our findings do not demonstrate a statistically significant association in this cohort. 

Although traditional markers such as serum creatinine and urea remain central to CKD staging, assessment of thyroid function may help identify subclinical thyroid dysfunction, which is common in advanced CKD and may contribute to fatigue, metabolic alterations, and cardiovascular risk. Recognizing these alterations may assist clinicians in differentiating CKD-related biochemical changes from primary endocrine disorders, thereby avoiding unnecessary interventions or misinterpretation of laboratory results. While our findings highlight an association between CKD stage and thyroid function alterations, the cross-sectional nature of the study precludes recommendations regarding routine monitoring. The use of the Cockcroft-Gault equation instead of the CKD-EPI equation for eGFR estimation may have influenced CKD staging accuracy and represents a methodological limitation of the present study. Further prospective studies are required to determine clinical benefit.
